# Psychosocial Work Factors, Job Stress and Strain at the Wheel: Validation of the Copenhagen Psychosocial Questionnaire (COPSOQ) in Professional Drivers

**DOI:** 10.3389/fpsyg.2019.01531

**Published:** 2019-07-02

**Authors:** Sergio A. Useche, Luis Montoro, Francisco Alonso, Juan C. Pastor

**Affiliations:** ^1^Development and Advising in Traffic Safety Research Group, University Research Institute on Traffic and Road Safety, University of Valencia, Valencia, Spain; ^2^FACTHUM.lab (Human Factor and Road Safety) Research Group, University Research Institute on Traffic and Road Safety, University of Valencia, Valencia, Spain

**Keywords:** psychosocial work factors, work environment, job stress, COPSOQ, professional drivers, transport workers

## Abstract

**Introduction:**

Psychosocial work environment has been related to many negative health outcomes in different workforces. However, evidence in this regard is still limited in the case of transport workers, and most of the tools used in research, often excessively generic, do not fully consider the specific key stressors, and adverse issues present in the psychosocial environment of professional driving.

**Objective:**

Thus, the purpose of this study was to obtain a complete description of the validation of measurement applied to psychosocial factors at work in professional drivers, using the Enterprise version (2018) of COPSOQ-III.

**Methods:**

The data was collected from 726 Spanish professional drivers, and the analyses were conducted using the competitive Confirmatory Factor Analysis or CFA, obtaining basic psychometric properties and an optimized structure for the instrument applied to active transport workers.

**Results:**

The results suggest a clear factorial structure, high factorial weights, internal consistency, and an improved adjustment to the psychosocial conditions of this group, excluding a set of items with low psychometrical adjustment and keeping the five-factor structure of the questionnaire: demands, influence and development, interpersonal relationships and leadership, job insecurity, and strain-effects and outcomes.

**Conclusion:**

Overall, what was found in this study supports the hypothesis that the validated version of COPSOQ in professional drivers, together with complementary information sources specific for their work environment, may have a relevant research value and some important practical implications for the improvement of the occupational safety, and health within the typically vulnerable industry of transportation.

## Introduction

Typically, psychosocial risks constitute a component that is both frequent and problematic in all occupations, and their impact on the health, safety and welfare of employees has been scientifically documented during the last decades (Leka et al., 2015; Bliese et al., 2017). Furthermore, different policies in the occupational field, especially those using evidence-based diagnosis and interventions, show a relative success in preventing adverse outcomes that may affect the job quality of the working population. However, there are certain occupational groups that the applied evidence calls “vulnerable,” given the high amount of adverse conditions under which they perform job-related tasks and their consequent translation into negative outcomes such as occupational diseases, low performance, and accidents at work. One of these groups is, undoubtedly, the collective of professional drivers (Lemke and Apostolopoulos, 2015; Cendales et al., 2016).

To say it shortly, psychosocial risks at work can be understood as features of the task design as well as the social, organizational, and management settings of the job that could potentially derive in the physical or psychological harm of workers, usually accompanied by changes in their feelings, attitudes, behavior, and in their physiological functions (Cox and Griffiths, 2005). In the particular case of professional drivers, the evidence has systematically demonstrated how the inherent adverse, and pressing working conditions that exist in their job environment, such as the excessive amounts of demands (i.e., time pressure, long, and irregular work schedules) and a considerably low skill discretion. These are associated with several risks and consequences for the health and welfare of this occupational group (Tse et al., 2006; Santos and Lu, 2016; Useche et al., 2018). Hege et al. (2019) and Sturm et al. (2019) have also shown how adverse working conditions have been associated with adverse consequences in different spheres, such as physical and mental health, job performance, and occupational safety outcomes. In the particular case of transport workers, frequent conditions such as time pressure, difficult weather conditions, environmental overstimulation, and shift-work increase even more the risk of (e.g., performing risky road behaviors and suffering severe crashes involving injured or fatal victims Ba et al., 2018; Gómez et al., 2018).

In short, health outcomes of professional drivers working under highly demanding conditions can be summarized in terms of both physical and mental illnesses, such as: ergonomic complications associated with physical working conditions (Abledu et al., 2014; Jadhav, 2016; Useche et al., 2018), hypertension (Hirata et al., 2012; Djindjić et al., 2013; Platek et al., 2017), respiratory and gastrointestinal disorders (Ekpenyong et al., 2012; Ronchese and Bovenzi, 2012), eye problems (Murray et al., 2017), lung cancer related to the prolonged exposition to contaminant and toxic gasses (Zuskin et al., 1994; Tsoi and Tse, 2012), metabolic syndrome (Lemke et al., 2017; Hege et al., 2019), sleep problems and chronic fatigue (Sabbagh-Ehrlich et al., 2005; Braeckman et al., 2011; Tellez López et al., 2015; Useche et al., 2017; Garbarino et al., 2018), psychological distress and several mental health disturbances such as anxiety, and depressive disorders (Narciso and Mello, 2017; Unsworth et al., 2017; Davidson et al., 2018). Other applied researches, such as the one performed by Taylor and Dorn (2006), Tse et al. (2006), Chung and Wong (2011), Useche et al. (2018, 2019) and Pérez-Fuentes et al. (2019) have related adverse working conditions, workplace stress and burnout of various occupational groups to both adverse psychological health indicators (such as the psychological distress measured by the short form of Goldberg’s GHQ-12) and negative lifestyle outcomes. These contribute to the development of further health issues potentially explaining scenarios of job losing, absenteeism, sick leave, and/or disability.

Although workplace causalities are decreasing worldwide, as a result of diverse regulations, interventions and improvements in industrial safety, occupational accidents in the industry of transportation are still considered a challenging issue in many countries, including Spain (Boada-Grau et al., 2012b; Montoro et al., 2018). Following an applied perspective, the task of reducing the number of work injuries largely depends on the available knowledge on their causal factors and dynamics, and this remains a pending issue for both researchers and road safety practitioners, considering that relatively little is known of psychosocial factors related to the work environment that may explain occupational accidents suffered by professional drivers (Gamero et al., 2018).

Research in psychosocial factors at work involves different possible procedures to retrieve information from the working population in different spheres, and it may involve both qualitative, quantitative, and mixed methods (Ballard et al., 2004; Institute of Medicine and National Research Council (Us) Committee on the Review of Niosh Research Programs, 2009; Iavicoli et al., 2015). One of the most used is, nowadays, the self-report questionnaire method; it offers, apart from the possibility of obtaining useful descriptive data on the work environment, the chance to collect further information about occupational conditions and perceived factors related to the occupational settings, such as job satisfaction, fatigue, social support and work stress -under different approaches. Among those, the Job Demand-Control (JDC; Karasek, 1998) and the Effort-Reward Imbalance (ERI; Siegrist, 2002) models are the most addressed in the empirical literature for what concerns several working groups, including professional drivers. Nevertheless, and although the approaches mentioned for the study of work-related stress offer a valuable both qualitative and quantitative source of data for occupational research, the progressive development of the Copenhagen Psychosocial Questionnaire (COPSOQ; Kristensen et al., 2005) represented an undeniable advance in the study of other factors that, together with job stress, may imply potential psychosocial risks for different groups of workers. In this regard, another undisputable advantage of this instrument is the potential comparability between different professional groups (Kristensen et al., 2005; Moncada et al., 2014; Nübling et al., 2014). Also, and overall, researchers agree on the fact that well-validated standardized questionnaires are essential instruments in the occupational research, since they are a necessary step for the design and implementation of practical preventive actions (Kristensen et al., 2005). In this regard, applied studies have supported the fact that questionnaires inquiring the perceived working conditions allow occupational researchers and managers to obtain more insights and relevant information than the management of crude administrative data, since the latter presents several methodological shortcomings (Sturm et al., 2019).

Nowadays, the COPSOQ questionnaire represents one of the most important standardized tools for the assessment of psychosocial risk factors at work, being translated into more than 25 different languages, generally finding good reliability, and internal consistency (Dicke et al., 2018). As for occupational groups covered by empirical studies that used the COPSOQ, several versions of the questionnaire, especially the COPSOQ-I (Kristensen et al., 2005) and COPSOQ-II (Pejtersen et al., 2010) have been already validated in several industries and occupational groups that are psychosocially considered as highly vulnerable, such as education workers (Dicke et al., 2018), technicians and administrative employees (Moncada et al., 2014), and service workers (Alvarado et al., 2009, 2012). However, although other similar tools have developed specific strategies for the measurement of psychosocial factors at work in commercial drivers, among which we find the ELBus-21 specifically designed for bus drivers (Boada-Grau et al., 2012a), and the development of specific questions in the Occupational Stress Index (Belkić, 2000; Belkić and Savić, 2008) stands out, it is worth mentioning that, to the date, there is no validated version of the COPSOQ for the specific case of professional drivers.

### Objective and Hypothesis of the Study

The purpose of this study was to describe, in detail, the validation of measurements of psychosocial work-related factors of professional drivers using the Nubling’s version of COPSOQ-III. It is hypothesized that, given the proven reliability, consistency and validity of the Copenhagen Psychosocial Questionnaire, and its adaptability to different occupations, the confirmatory model based on the five root factors of the instrument will present a good fit and optimal factor loadings. However, slight factor variations in the structure of the instrument may take place, especially bearing in mind some key features of the working environment and task-design of professional drivers, such as the expected low variability in skill discretion/degree of freedom and influence at work.

## Materials and Methods

### Sample

For this research, we used a representative sample of 726 professional drivers from the 17 autonomous communities (regions) of Spain. Regarding gender, and as it was expected when considering the overrepresentation of men in this workforce, 98.6% of them were males and 1.4% females. The mean age of the sample was *M =* 47.10 (*SD* = 8.05) years of age, ranging between 24 (minimum), and 70 (maximum). The final database included both cargo (goods) and passenger drivers: 31 (4.4%) city bus drivers; 121 (17.1%) inter-urban bus drivers; 486 (68.5%) long-haul and freight-vehicle drivers; 57 (8%) van/small truck drivers, and 31 (2.0%) were driving other types of vehicles.

The mean hourly intensity of driving was *M =* 7.82 (*SD* = 1.92) hours/day and the average number of days driving was *M =* 5.23 (*SD* = 0.69) days/week. As for road safety records, the mean accident rate during the last 2 years was *M =* 0.40 (*SD* = 1.04), and the average amount of traffic fines received was *M =* 1.15 (*SD* = 2.04).

### Study Design and Procedure

For this (cross-sectional) empirical research, participants completed a paper-based self-report questionnaire. As a part of a large collaborative research project with transport companies and associations of professional drivers in Spain, participants were directly invited to take part in the study by their different Spanish transport companies; thus, they were selected through a convenience (non-probabilistic) sampling method. The contact and operational discretion in cooperation with the transport companies and the processes of data gathering took us around 5 months of time. All commercial drivers were asked to complete the questionnaire during approximately 1 h of their formation modules, as previously agreed by companies as a contribution for our research. A research assistant was always with the participants during the completion of the instrument. Participants were informed about the protection of their personal data by means of an informed consent form, which highlighted that the data would be exclusively used for research purposes. The general response of the study rate was 81%.

### Description of the Questionnaire

We used the Standard Enterprise Version -updated for the year 2018- of the COPSOQ-III; Nübling et al., 2018), a self-report instrument for measuring psychosocial factors at work based on the original version by Kristensen et al. (2005). Our instrument is Kristensen, Hannerz, Hegh and Borg’s short version, and it was developed and adapted by Nübling et al. (2006) through a wide generic sample of German workers. Overall, this adapted version presents several advantages over the original questionnaire, such as high succinctness, consistency and reliability; moreover, it introduces some additional scales in replacement of short scales/supplements of the original COPSOQ that are copy-righted (and, therefore, cannot be freely used by researchers and occupational health practitioners). Although other COPSOQ forms had been previously adapted to the Spanish language (see Moncada et al., 2014), this specific version of the instrument had not been previously translated into Spanish. Thus, it was systematically translated by a set of experienced practitioners in the areas of modern languages and occupational research, carrying out an ulterior re-translation to English, to ensure that the Spanish version was pertinent enough, and presented a high coherence with the original scale. Additionally, and following what was suggested by the authors of the employed version of the questionnaire (Nübling et al., 2006, 2018), some specific questions related to the work environment, and conditions of professional drivers (e.g., hourly intensity, shift distribution, and occupational accidents) were added for further analyses. The final version of the questionnaire was given to participants in Spanish and had two main sections.

The first one inquired about: (a) individual and demographic variables (e.g., gender, age, education, town or city of residence, and current occupation), and (b) driving-related variables, such as type of vehicle(s) driven at work, transport modalities (i.e., passengers, cargo, and other), hours driving/day, days driving/week, shift-working, stability of time schedules, and road safety indicators (i.e., traffic crashes suffered and fines perceived along the last 2 years during work schedules).

The second part of the instrument was composed of the “statements on work and activity” initially presented by Nübling et al. (2006) in the year, and also summarized in the *enterprise version* (released in 2018) of the German adaptation of the COPSOQ. The full questionnaire of this version has a total of 74 items sub-divided into five main factors, to be answered using a Likert scale that varies according to the question form (e.g., frequency: “*how often…?*,” level of agreement: “*to what extent would you say that…?*”). In Table 1, specific components/structure of the factors and item numbers are detailed. Finally, the 12-item version of the General Health Questionnaire (GHQ-12; Goldberg, 1992) was used in order to test the convergent validity of COPSOQ factors, based on the same theoretical considerations and relationships between psychosocial factors at work and psychological health presented in the introduction of this paper. This short Likert scale is composed of 12 questions aimed at assessing different potential symptoms that may have affected the subject’s mental health in the form of *psychological distress* during the previous month.

**TABLE 1:** Factor composition and detail of items/sub-scales of the analyzed version of the questionnaire.

**#^a^**	**Sub-scale**	**N (items)**	**Questions**
**F1: Demands (16 items)**			
1	Quantitative demands	4	B1: 1–4
2	Emotional demands	3	B1: 5–7
3	Demands for hiding emotions	2	B1: 8–9
4	Work-privacy conflict	7	B2: 1–7
**F2: Influence and development (12 items)**			
5	Influence at work	3	B3: 1–3
6	Degree of freedom at work	2	B3: 4–5
7	Possibilities for development	3	B4: 1, B5: 1–2
8	Meaning of work	2	B5: 3–4
9	Workplace commitment	2	B5: 5–6
**F3: Interpersonal relations and leadership (22 items)**			
10	Predictability	2	B6: 1–2
11	Role clarity	3	B6: 3–5
12	Role conflicts	3	B6: 6–8
13	Quality of leadership	4	B7: 1–4
14	Social support	4	B8: 1–4
15	Feedback	2	B8: 5–6
16	Social relations	1	B8: 7
17	Sense of community	2	B8: 8–9
18	Mobbing	1	B8: 10
**F4: Job insecurity (6 items)**			
19	Job Insecurity	6	B9: 1–6
**F5: Strain (effects, outcomes) (18 items)**			
20	Intention to leave	2	B10: 1–2
21	Job satisfaction	7	B11: 1–7
22	General health	1	B12: 1
23	Energy and mental wellbeing	5	B13: 1v5
24	Fatigue	3	B14: 1–3
	Total	74	–

*^*a*^Original subscale of the item.*

### Ethics Statement

To perform this research, the Social Science in Health Research Ethics Committee of the University of Valencia was consulted; it confirmed that the study responded to the general ethical principles, which are now necessary to research in Social Sciences, and it certified its accordance with the Declaration of Helsinki. Thus, the study received a favorable opinion and the researchers were authorized to carry it out (IRB approval number H1517828805528). Moreover, we employed an Informed Consent Statement containing ethical principles and data treatment details (100% of participants provided their written and informed consent), in which the objective of the study, the approximate duration of the survey, the treatment of the personal data and the voluntary participation were explained, and participants received it before completing the questionnaire. The research did not use personal and/or confidential data, and the involvement was anonymous, in order to avoid any potential risk for the integrity of the study contributors.

### Data Processing (Statistical Analysis)

First of all, data curation and descriptive analyses (means, standard deviations, and other basic measures) were performed. Then the factorial structure of the COPSOQ-III was, after an initial assessment via exploratory factor analysis or EFA with maximum likelihood (a statistical method used to uncover the underlying structure of a relatively large set of variables), tested by means of a rigorous confirmatory procedure. We did this considering model fit indexes and reliability/consistency scores, as it is suggested by other studies measuring similar issues in occupational and organizational settings (Boada-Grau et al., 2012b; Moncada et al., 2014). More specifically, competitive confirmatory factor analyses (CFA) were assessed and subsequently tested. The research employed confirmatory factor models, considering that this scale had already been tested with other samples from different working populations, and so both theoretical and empirical models were available. This constituted the *a priori* or initial model to be contrasted (see section “Structural Models” for model specifications). CFA also implies some advantages in relation to the treatment of missing data, and to categorical and non-normal variables (Finney and DiStefano, 2013). These CFAs were specified and estimated in SPSS AMOS (version 24.0). Weighted least square mean and variance corrected (WLSMV) estimation were utilized, since the data were ordinal and did not meet the assumption of multivariate normality.

As recommended in the specialized literature, the model fit was assessed by means of several statistics, and indices from different logics and families (Marsh et al., 2004). In this specific case, all available types of indices for the method of estimation were employed: chi-square, CFI, and RMSEA. Fit was established based on the cut-off criteria suggested by Marsh et al. (2004): a CFI higher than 0.90 (better if higher than 0.95) and RMSEA lower than 0.08 were indicative of an adequate model fit. Anyhow, the acceptability of the model was also assessed using the strength and interpretability of the estimates of the parameters and through the absence of large and substantially adequate indices of modification. The reliability (or internal consistency) of the scale and the items were also assessed through: (1) alpha coefficients (α), and (2) the composite reliability index (CRI), a complementary consistency index statistically based on the factor loadings, and residuals in the confirmatory results, that overcomes some of the shortcomings of alpha as an estimate of reliability (Raykov, 2001; Raykov and Marcoulides, 2011). Also, the convergent validity was tested using the indicator of psychological distress provided by Goldberg’s GHQ-12. Finally, comparative analyses were conducted after the categorical variables were defined, by means of parametrical tests for mean comparison (Student’s *t*-test for independent samples), which were also carried out using the SPSS AMOS software.

## Results

### Structural Models

**FIGURE 1:**
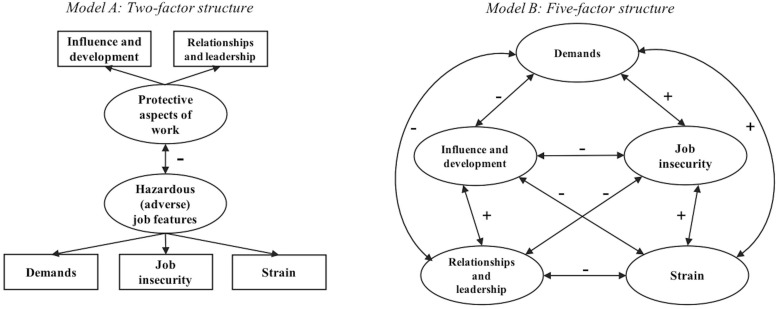
Model specifications for competitive confirmatory factor analysis. *Model A* (left) hypothesizes a two-factor structure based on the directionality of the associations among items, and the hypothesized *Model B* (right) follows the original structure of COPSOQ-III.

With the purpose of understanding the factorial structure of the Spanish version of the COPSOQ-III scale, factor analyses were carried out. An initial exploratory factor analysis (EFA) showed that the scale could be adjusted to five dimensions with acceptable factor loadings and a relatively high correspondence with the items originally composing the theorized factors or dimensions. Therefore, and as the dimensionality of the measuring items of COPSOQ had been previously studied and supported with previous applications in the working population, we proceeded to carry out the confirmatory analysis. Thus, two competitive CFAs were tested, specified as follows: (a) the original structure with five root factors, and (b) a two-factor structure, which is the most economical (simple and parsimonious, but strict) solution for a scale that represents covariability among the items, based on the aggrupation of adverse/risky psychosocial features of the job (factors 1: *demands*, 4: *job insecurity*, and 5: *strain*) and protective/non-protective aspects at work (factors 2: *influence and development*, and 3: *interpersonal relationships and leadership*). The specifications of the models used for the competitive CFA procedure and the hypothesized directionality of their associations are shown in Figure 1.

The model fit for the two-factor model was substantially inadequate: *x*^2^(2554) = 18979.679, *p <* 0.001, RMSEA = 0.094 with 90% CI of 0.093–0.095, CFI = 0.487, NFI = 0.452, and CMIN/DF = 7.431. Furthermore, the model fit for the *a priori* five factor model was: *x*^2^(2545) = 15364.495, *p <* 0.001, RMSEA = 0.083 with 90% CI of 0.082–0.085, CFI = 0.600, NFI = 0.557, and CMIN/DF = 6.037. Neither the five-factor structure nor the two-factor solution reasonably and adequately fitted the data, but it was clear that the five-factor model was much better. A close analysis of the five-construct model, with better fit indexes, showed that an amount of items were not significantly related to the factors they should have theoretically belonged to (*p >* 0.05). Considering that the original instrument has 74 items for measuring the aforementioned five main variables or factors, we decided to clear the scale by excluding those items which reported obvious psychometric issues in the measurement of their respective constructs, including those items with factorial loadings *(λ)* under 0.50. Accordingly, a total of 22 items were dismissed.

The new 5-factor structure for the outstanding 52 items was tested. This modified and simplified model fitted the data considerably well: *x*^2^(1169) = 2436.905, *p <* 0.001, RMSEA = 0.039 with 90% CI of 0.037–0.041, CFI = 0.947, NFI = 0.903, and CMIN/DF = 2.085. It is relevant to notice how, when we compare this model fit to a two-factor solution with these items, the five-factor structure presents a much better fit, as the fit indices for the two-factor solution are: *x*^2^(1184) = 4456.125, *p <* 0.001, RMSEA = 0.063 with 90% CI of 0.061–0.064, CFI = 0.861, NFI = 0.822, and CMIN/DF = 3.765.

**FIGURE 2:**
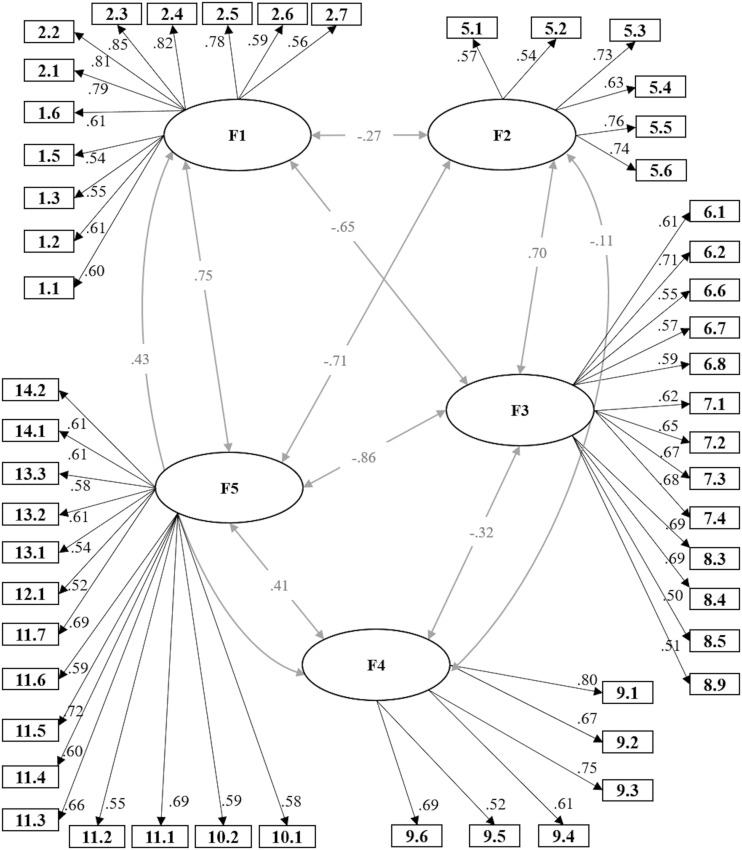
Standardized parameter estimates. All estimates were *p <* 0.001; the numbers within squares represent the original numbers of the items in the COPSOQ (as shown in Table 2).

Table 2 presents the descriptive content (average scores and standard deviations) of the items, the standardized factor loading of each one of them, their standard error and statistical significance, and the factor to which each item belongs. It is possible to see that all factor loadings are considerable, positive, and statistically significant in their respective constructs, as it is represented in Figure 2.

**TABLE 2:** Item content, factor that the item belongs to, standardized factor loading (λ), standard error (SE), and *p*-values in the retained model.

**Question**	**Item content**	**Sub-scale^a^**	***M***	***SD***	**λ**	**SE**	***P***
**F1: Demands**							
B1.1	Do you have to work very fast?	1	3.57	1.01	0.60	0.06	<0.001
B1.2	Do you work at a high pace throughout the day?	1	3.43	1.06	0.61	0.04	<0.001
B1.3	How often do you not have time to complete all your work tasks?	1	2.88	1.06	0.55	0.06	<0.001
B1.5	Do you have to do overtime / extra work?	2	3.14	1.31	0.54	0.10	<0.001
B1.6	Do you have to deal with other people’s personal problems as part of your work?	2	3.11	1.27	0.61	0.10	<0.001
B2.1	The demands of my work interfere with my home and family life	4	3.46	1.28	0.79	0.10	<0.001
B2.2	The amount of time my job takes up makes it difficult to fulfill my family responsibilities	4	3.44	1.29	0.81	0.10	<0.001
B2.3	My work drains so much of my energy that it has a negative effect on my private life	4	3.37	1.30	0.85	0.11	<0.001
B2.4	My work takes so much of my time that it has a negative effect on my private life	4	3.38	1.32	0.82	0.11	<0.001
B2.5	It happens that I should be at home and at work at the same time	4	3.10	1.32	0.78	0.10	<0.001
B2.6	I take care of work-related tasks outside of my working time as well	4	2.85	1.40	0.59	0.10	<0.001
B2.7	I am available in my free time for people with whom I deal professionally	4	2.66	1.41	0.56	0.10	<0.001
**F2: Influence and development**							
B5.1	Do you have the possibility of learning new things through your work?	7	2.96	1.06	0.57	0.05	<0.001
B5.2	Can you use your skills or expertise in your work?	7	3.23	1.06	0.54	0.06	<0.001
B5.3	Is your work meaningful?	8	3.76	0.98	0.73	0.09	<0.001
B5.4	Do you feel that the work you do is important?	8	3.81	1.00	0.63	0.08	<0.001
B5.5	Are you proud to be part of the company?	9	3.70	1.14	0.76	0.10	<0.001
B5.6	Do you enjoy telling others about your place of work?	9	3.14	1.23	0.74	0.11	<0.001
**F3: Interpersonal relationships and leadership**							
B6.1	At your place of work, are you informed well in advance concerning for example important decisions, changes, or plans for the future?	10	2.35	1.17	0.61	0.11	<0.001
B6.2	Do you receive all the information you need in order to do your work well?	10	3.06	1.02	0.71	0.06	<0.001
B6.6	Are contradictory demands placed on you at work? [−]	12	3.38	1.18	0.55	0.07	<0.001
B6.7	Do you sometimes have to do things, which ought to have been done in a different way? [−]	12	2.86	1.12	0.57	0.07	<0.001
B6.8	Do you sometimes have to do things, which seem to you to be unnecessary? [−]	12	2.91	1.18	0.59	0.08	<0.001
B7.1	Do your immediate superiors make sure that the individual member of staff has good development opportunities?	13	2.48	1.22	0.62	0.08	<0.001
B7.2	Do your immediate superiors give high priority to job satisfaction?	13	2.43	1.23	0.65	0.08	<0.001
B7.3	Are your immediate superiors good at work planning?	13	2.80	1.19	0.67	0.08	<0.001
B7.4	Are your immediate superiors good at solving conflicts?	13	2.77	1.22	0.68	0.08	<0.001
B8.3	How often do you get help and support from your nearest superior?	14	2.75	1.32	0.69	0.09	<0.001
B8.4	How often is your immediate superior willing to listen to your work-related problems?	14	2.97	1.40	0.69	0.09	<0.001
B8.5	How often do you talk with your superior about how well you carry out your work?	15	2.31	1.28	0.50	0.07	<0.001
B8.9	Is there good co-operation between your colleagues at work?	17	3.56	1.12	0.51	0.07	<0.001
**F4: Job insecurity**							
B9.1	Are you worried about becoming unemployed?	19	3.41	1.26	0.80	0.06	<0.001
B9.2	Are you worried about new technology making you / your work redundant?	19	2.83	1.28	0.67	0.05	<0.001
B9.3	Are you worried about it being difficult for you to find another job if you became unemployed?	19	3.25	1.21	0.75	0.05	<0.001
B9.4	Are you worried about being transferred to another job against your will?	19	2.56	1.21	0.61	0.05	<0.001
B9.5	Are you worried about the timetable being changed (shift, weekdays, time to enter and leave…), against your will?	19	2.90	1.26	0.52	0.05	<0.001
B9.6	Are you worried about a decrease in your salary?	19	3.50	1.27	0.69	0.05	<0.001
**F5: Strain (effects, outcomes)**							
B10.1	How often have you thought about giving up your profession?	20	2.53	1.30	0.58	0.10	<0.001
B10.2	How often have you thought about changing your job?	20	2.59	1.31	0.59	0.04	<0.001
B11.1	How pleased are you with your work prospects? [−]	21	2.58	0.95	0.69	0.06	<0.001
B11.2	How pleased are you with the people you work with? [−]	21	2.42	0.82	0.55	0.05	<0.001
B11.3	How pleased are you with the physical working conditions? [−]	21	2.74	0.95	0.66	0.06	<0.001
B11.4	How pleased are you with the way your group is run? [−]	21	2.62	0.84	0.60	0.05	<0.001
B11.5	How pleased are you with the way your abilities are used? [−]	21	2.65	0.88	0.72	0.06	<0.001
B11.6	How pleased are you with your salary? [−]	21	3.34	1.07	0.59	0.06	<0.001
B11.7	How pleased are you with your job as a whole, everything taken into consideration? [−]	21	2.72	0.90	0.69	0.06	<0.001
B12.1	How many points (0–10) do you give to your present state of health? [−]	22	2.59	1.84	0.52	0.11	<0.001
B13.1	How often do you feel physically exhausted?	23	2.78	0.90	0.54	0.05	<0.001
B13.2	How often do you feel emotionally exhausted?	23	2.74	0.96	0.61	0.06	<0.001
B13.3	How often did you feel worn out?	23	2.74	0.92	0.58	0.06	<0.001
B14.1	At my work, I am full of energy. [−]	24	2.35	0.86	0.61	0.05	<0.001
B14.2	I am enthusiastic about my work. [−]	24	2.37	0.96	0.61	0.06	<0.001

*^*a*^Original subscale of the item.*

### Factor Correlations and Convergent Validity

Bivariate correlations between pairs of factors were, as it was hypothesized, statistically significant and considerably large. Association trends were grouped in two sets of factors: on one hand, factors 1 (demands), 4 (job insecurity), and 5 (strain), positively correlated among them and, on the other hand, factors 2 (influence and development), and 3 (interpersonal relationships and leadership) also presented a positive association between them. As it was initially hypothesized, both sets of factors were negatively and significantly correlated, as shown in Table 3, in which association (Pearson) coefficients (σ) are also available.

**TABLE 3:** Bivariate correlations (Pearson) between study factors.

**Factor**	**F1**	**F2**	**F3**	**F4**	**F5**	**CV^a^**
F1: Demands	1					
F2: Influence and development	–0.273^∗∗^	1				
F3: Interpersonal relationships and leadership	–0.655^∗∗^	0.703^∗∗^	1			
F4: Job Insecurity	0.433^∗∗^	–0.109^∗∗^	–0.323^∗∗^	1		
F5: Strain (effects, outcomes)	0.755^∗∗^	–0.712^∗∗^	–0.863^∗∗^	0.408^∗∗^	1	
CV^a^: Psychological distress	0.491^∗∗^	–0.439^∗∗^	–0.503^∗∗^	0.265^∗∗^	0.646^∗∗^	1

*^*a*^Introduced as a criterion variable (CV). ^∗∗^Correlation is significant at the 0.01 level (2-tailed).*

Furthermore, the convergent validity was evaluated by means of the correlation coefficients found between each one of the scores of the five resulting dimensions of COPSOQ after CFA and the psychological distress indicator provided by Goldberg’s GHQ-12, the latter used as a criterion variable (CV). In this regard, and following the hypothesized directions of the Pearson’ association coefficients, we found positive and significant correlations between psychological distress and factors 1 (demands; σ = 0.491), 4 (job insecurity; σ = 0.265), and 5 (strain; σ = 0.646). On the other hand, negative and significant correlations were found when crossing psychological distress with factors 2 (influence and development; σ = −0.439) and 3 (interpersonal relationships and leadership; σ = −0.503).

### Internal Consistencies

Alpha estimates were all above the usual 0.7 criteria, suggested by methodological sources (Morera and Stokes, 2016), that indicate adequate internal reliability: 0.919 for Demands; 0.854 for Influence and development; 0.911 for Interpersonal relationships and leadership; 0.852 for Job insecurity; and 0.901 for Strain. The composite reliability indices (CRI) had very satisfying reliabilities for all the latent constructs. CRI for F1 (Demands) was 0.983. The CRI for F2 (Influence and development) was 0.970. For F3 (Interpersonal relationships and leadership), CRI was 0.984. For F4 (Job insecurity) it was 0.981, Finally, CRI for F5 (Strain – effects and outcomes) it was 0.989.

## Discussion

This empirical research pursued the main objective of presenting the validation of the 74-item version of the COPSOQ (Nübling et al., 2006, based on the original questionnaire of Kristensen et al., 2005) in professional drivers. Overall, the results shown by this study confirmed that, in accordance with both the exploratory assessment and the previous theoretical and empirical background supporting the structure of the COPSOQ, the questionnaire keeps a well-adjusted configuration of five factors, which in turn keeps the five scales measured by the COPSOQ-III (Nübling et al., 2018), and guarantees a major psychometric and methodological value for measuring psychosocial factors at work, in this case focusing on professional drivers.

For what concerns the validity of the root COPSOQ-III factors, during the competitive analysis the five-factor model used through the CFA was significantly better (more succinct, parsimonious, and consistent) than the alternative solution that had been previously analyzed, considering: (a) the high concordance of the original structure with the resulting (validated) scale, with a relatively small loss of items, even bearing in mind that only items with λ coefficients (factor loadings) over 0.50 were considered adequate for the validated version of the instrument; (b) the high reliability/internal consistency coefficients (i.e., Cronbach’s Alphas and CRI indexes) obtained; (c) the satisfactory model fit, supported by the different indexes with optimal values (RMSEA = 0.039, CFI = 0.947, NFI = 0.903, and CMIN/DF = 2.085), in accordance with what has been already stated in the specialized literature on this topic (see Marsh et al., 2004 for further information); and (d) the coherence between the deleted items and the lack of variability observed in work settings such as the degree of freedom at work, that are commonly observed in professional driving (Tse et al., 2006; Chung and Wu, 2013; Gómez et al., 2018).

Also, the five main constructs measured by means of the instrument showed significant and coherent correlation sizes and directions, when compared with both the theoretical background of the questionnaire and with several other versions/applications of it (Kristensen et al., 2005; Aminian et al., 2017; Berthelsen et al., 2018). Also, there was a good coherence in the significant associations observed between the five factors of the scale and the psychological health indicator provided by the Goldberg’s GHQ-12 (i.e., *psychological distress*), used as a CV for assessing convergent validity.

### The Shortened Version of COPSOQ: Understanding the Work Environment of Professional Driving

After describing a set of factors that quantitatively support the validity and pertinence of the instrument in its current 52-item shortened version, some statistical-based variations should be qualitatively discussed. Firstly, out of the 22 eliminated items contained in the Nübling’s version of the instrument, 17 were items that, once excluded, just partially decreased the size of the original sub-scale; 5 other items with poor adjustment (3 for the sub-scale “influence at work,” and 2 for “degree of freedom at work”) constituted the full sub-scale, reason why they were deleted from the final version. This may have a plausible qualitative explanation: as it is described in other empirical researches dealing with this specific population, uniform, and scarcely variable outcomes of low skill discretion, decision making, and promotion prospects have been found in this type of jobs; in other words, some studies have shown how professional drivers have, generally, very little influence at work and therefore experience a substantially limited degree of freedom (Chung and Wu, 2013; Gómez et al., 2018). This is summed to other negative issues similarly observed with high frequency in professional drivers, such as job dissatisfaction and insecurity (Murray et al., 2017; Tran and Sokas, 2017), and elevated amounts of physical and psychological demands (Cendales et al., 2016), thus remarking the high psychosocial and environmental vulnerability of the workers who belong to the transport industry. However, and as it has been addressed in this paper, the role of generic scales for assessing the psychosocial work environment of specific occupations remains relatively limited in addressing in-depth particular issues in spheres such as ergonomics, task design and safety outcomes. In this regard, the authors of both the root-questionnaire used in this research and some other studies have suggested adding additional items, aimed at describing those settings that are particular of each working population, thus enhancing the qualitative interpretability of the findings of occupational studies carried out through this kind of tools (Nübling et al., 2006, 2018; Hege et al., 2018; Useche et al., 2018).

Finally, it is worth highlighting the need and importance of evidence-based diagnosis and intervention in psychosocial factors at work and adverse working conditions. These have been proved to be critical strategies for strengthening the proper identification and management of both individual and collective occupational risk factors present in the work environment of professional drivers, that also involves their job performance, safety, physical and mental health, and well-being (Tse et al., 2006; Hashemi Nazari et al., 2017).

## Conclusion

What was found in this study supports the hypothesis that the COPSOQ-III can have relevant applications in the improvement of occupational and road safety, by means of the study of both psychosocial work environment and psychosocial work factors which affect professional drivers. Furthermore, the validated tool, together with the use of complementary questions aimed at addressing the work particularities of the job of professional drivers (non-generic issues such as hourly intensity, schedule regularity and distribution, and health outcomes), can be particularly useful for implementing evidence-based occupational programs focused on the identification and subsequent intervention of those adverse working psychosocial conditions that affect this vulnerable occupational group.

## Limitations of the Study

Although in this research the sample was considerably large, the statistical parameters and model fit coefficients were satisfactorily tested, and the quality of the instruments had been previously supported by the empirical background, some methodological biasing sources should be considered. First, this study was performed using exclusively self-report-based data collection methods. In this regard, the evidence has shown how self-report measures can imply biases such as acquiescence (i.e., the total agreement of participants with the presented questions), social desirability and insincerity. Additionally, positive/negative affect may influence the participants’ style of response and the actually observed driving safety outcomes (Af Wåhlberg, 2010; Brenner and DeLamater, 2016; Chai et al., 2016). Therefore, we suggest, for future researches and interventions in the field, performing further statistical procedures such as stochastic frontier estimations (SFE), and considering the inclusion of supplementary scales intended to measure and control potential biasing sources that may influence the results of predictive and/or explanatory studies (Rosenman et al., 2011; McCullagh and Rosemberg, 2015).

Regarding the tool used for collecting the data, it is worth emphasizing that, although standardized scales and research tools -such as COPSOQ- have a proven value for what concerns the measurement of psychosocial work-related factors across different occupational groups, they fail to address potential specific stressors and hazardous working conditions particularly affecting each one of them. Thus, and as it was highlighted by Nübling et al. (2006, 2018), it is suggestible to assess specific stressors and issues related to the work structure of each occupational group, including additional short scales and/or questions that may enhance the interpretation and crossing of results. Finally, it is worth remembering that the current (cross-sectional) study used a single measurement for the data collection; in this regard, and although it may involve higher costs and more time, researchers are encouraged to collect longitudinal data, since it allows for the conduction of invariance tests of the instruments over time.

## Data Availability

The raw data supporting the conclusion of this manuscript will be made available by the authors, without undue reservation, to any qualified researcher.

## Ethics Statement

In order to conduct this research, the Social Science in Health Research Ethics Committee of the University of Valencia was consulted; it confirmed that the study responded to the general ethical principles, which are now necessary to research in Social Sciences, and it certified its accordance with the Declaration of Helsinki. Thus, the study received a favorable opinion and the researchers were authorized to carry it out (IRB approval number H1517828805528). Moreover, we employed an Informed Consent Statement containing ethical principles and data treatment details (all participants provided their written and informed consent), in which the objective of the study, the approximate duration of the survey, the treatment of the personal data, and the voluntary participation were explained, and participants received it before completing the questionnaire. The research did not use personal and/or confidential data, and the participation was anonymous, thus avoiding any potential risks for the integrity of the participants.

## Author Contributions

SU conceived and designed the research. SU and LM collected the data, wrote, and revised the manuscript. SU and FA analyzed the data. FA contributed to the reagents, materials, and analysis tools. JP conceived and revised the manuscript.

## Conflict of Interest Statement

The authors declare that the research was conducted in the absence of any commercial or financial relationships that could be construed as a potential conflict of interest.
